# Therapeutic Management of Schistosomiasis in Pregnancy: A Comprehensive Review of Praziquantel Safety and Clinical Outcomes

**DOI:** 10.3390/biomedicines14092018

**Published:** 2026-09-08

**Authors:** Hamid Mn Mustafa, Tahani Elfaki, Ishag Adam

**Affiliations:** 1 Department of Pharmacy Practice, College of Pharmacy, Qassim University, Buraydah 51452, Saudi Arabia; ha.mustafa@qu.edu.sa; 2 Clinical Pharmacology Department of Pharmacology, Faculty of Pharmacy, University of Gezira, Wad Madani 21111, Sudan; tahani2007@uofg.edu.sd; 3 Department of Obstetrics and Gynecology, College of Medicine, Qassim University, Buraydah 51452, Saudi Arabia

**Keywords:** praziquantel, pregnancy, schistosomiasis, maternal health, fetal safety, mass drug administration

## Abstract

**Background/Objectives:** Schistosomiasis affects about 250 million people globally, including an estimated 40 million women of reproductive age and over 10 million pregnant women each year. Praziquantel is a key component of schistosomiasis control and is recommended by the World Health Organization (WHO) for use during pregnancy; nevertheless, its use is uneven due to ongoing safety concerns. **Methods:** This narrative review synthesizes evidence from randomized controlled trials (with the strongest evidence for the second and third trimesters), observational studies (including limited first-trimester exposures), pharmacokinetic analyses, case series, and WHO policy documents published between 1980 and March 2026. **Results:** Across all major Schistosoma species, praziquantel demonstrates high efficacy in pregnancy, with cure rates comparable to those in non-pregnant populations. No increased risk of miscarriage, stillbirth, congenital anomalies, preterm birth, or low birth weight has been observed in randomized controlled trials (RCTs) or cohort studies. Treatment of *S. haematobium* improves maternal anemia and may confer benefits on neonatal iron status and immune development; evidence gaps remain regarding first-trimester exposures and long-term offspring outcomes. **Conclusions:** Aligning national policies with WHO guidance and integrating praziquantel into antenatal care could substantially reduce maternal morbidity and improve neonatal health.

## 1. Introduction

### 1.1. Background

Schistosomiasis is a chronic parasitic disease caused by trematodes of the genus Schistosoma and remains a major contributor to morbidity in low- and middle-income countries. Globally, more than 250 million people are infected, with over 700 million at risk of exposure, particularly in sub-Saharan Africa, Southeast Asia, and parts of the Middle East and South America [1,2,3]. Women of reproductive age constitute a substantial proportion of this burden, with an estimated 40 million infected annually and more than 10 million experiencing infection during pregnancy [1,3,4].

Despite this considerable burden, pregnant women have historically been excluded from schistosomiasis treatment programs. Early concerns regarding teratogenicity, combined with limited pregnancy-specific clinical data and risk-averse regulatory practices, led to the routine exclusion of pregnant women from mass drug administration (MDA) campaigns. This exclusion persisted even as praziquantel became widely used and extensively studied in non-pregnant populations.

Praziquantel, introduced in the late 1970s, is a pyrazinoisoquinoline derivative and remains the only WHO-recommended treatment for all major human *schistosome* species, including *S. mansoni*, *S. haematobium*, and *S. japonicum* [5,6,7]. According to the current U.S. Food and Drug Administration (FDA) Pregnancy and Lactation Labeling Rule (PLLR), available evidence does not indicate an association between praziquantel use during pregnancy and an increased risk of major congenital malformations, miscarriage, or adverse maternal or fetal outcomes. This conclusion is supported by two randomized controlled trials involving pregnant women treated for schistosomiasis (one against *S. mansoni* in Uganda [8] and the other against *S. japonicum* in the Philippines [9]), as well as retrospective observational studies, case series, and case reports, all of which found no significant differences in birth weight, fetal death, perinatal mortality, major birth defects, or other adverse pregnancy outcomes compared with controls [10]. Furthermore, animal reproduction studies in rats and rabbits demonstrated no evidence of developmental toxicity or fetal harm at doses up to 0.65- and 1.3-fold, respectively, the maximum recommended human daily dose (75 mg/kg/day, based on body surface area). Regarding lactation, limited evidence indicates that praziquantel is excreted into human breast milk in low concentrations; however, there are insufficient data on its effects on breastfed infants or milk production. Therefore, the benefits of breastfeeding should be weighed against the mother’s clinical need for praziquantel and any potential risks to the infant [11]. Based on accumulating safety data, WHO recommends praziquantel treatment during pregnancy but explicitly recommends treatment after the first trimester (recommend treatment only during 2nd and 3rd trimesters) in the 2022 guideline; this recommendation was reaffirmed in subsequent guidelines [12,13,14].

Nevertheless, implementation of these recommendations remains inconsistent. Many national control programs continue to exclude pregnant women from MDA campaigns, citing outdated guidelines, concerns among healthcare workers, and sociocultural perceptions surrounding medication use during pregnancy [10,15,16]. This policy–practice gap results in millions of untreated infections annually, perpetuating maternal morbidity and undermining schistosomiasis control and elimination efforts.

Schistosomiasis during pregnancy is associated with a range of adverse maternal outcomes, including anemia, chronic inflammation, urogenital pathology, and impaired nutritional status [2,17,18,19]. These maternal effects may indirectly influence fetal growth and neonatal health, contributing to low birth weight, intrauterine growth restriction, and early childhood anemia [20,21,22,23]. Consequently, the question of praziquantel use in pregnancy is not solely a pharmacological issue but a critical maternal–fetal health concern.

### 1.2. Novelty and Rationale of This Review

Although previous reviews and WHO technical reports have addressed praziquantel use during pregnancy, no recent narrative review has comprehensively integrated the expanding body of evidence published over the past decade. This review is novel in several respects. First, it synthesizes all available data through March 2026, including recent randomized controlled trials, such as the free BILy Gabon study [24], and updated pharmacokinetic analyses in pregnant and lactating women [25]. Second, it integrates clinical efficacy and safety data with emerging evidence on placental involvement, offspring immune modulation, and early life outcomes. Third, it critically examines the persistent disconnect between WHO recommendations and national implementation, incorporating qualitative evidence on patient and provider perceptions. Finally, this review frames praziquantel treatment during pregnancy within a life-course and maternal-health perspective, emphasizing benefits that extend beyond parasitological cure.

By consolidating pharmacological, clinical, immunological, and policy-level evidence, this review aims to provide the most up-to-date and comprehensive assessment of praziquantel use during pregnancy and to offer evidence-based guidance for clinicians, researchers, and policymakers.

## 2. Materials and Methods

### 2.1. Overall Approach

This study employed a structured, transparent, non-systematic narrative review methodology, guided by the Scale for the Assessment of Narrative Review Articles (SANRA). The objective was to capture and integrate the full spectrum of evidence relevant to praziquantel use during pregnancy, acknowledging the heterogeneity of study designs and outcomes in this field.

### 2.2. Literature Search Strategy

A comprehensive literature search was performed through PubMed/MEDLINE, Scopus, Web of Science, Embase, and Google Scholar. The search covered publications from January 1980 to March 2026. Search terms were combined using Boolean operators and included the following: “praziquantel”, “schistosomiasis”, “bilharzia”, “pregnancy”, “maternal health”, “fetal safety”, “birth outcomes”, “pharmacokinetics”, “mass drug administration”, and “WHO guidelines”. Additional sources included WHO technical reports, policy documents, Cochrane and Campbell systematic reviews, and reference lists of key articles to identify further relevant studies. Examples of Boolean strings used: (praziquantel AND pregnancy), (praziquantel AND schistosomiasis AND pregnancy), (praziquantel AND pregnancy AND safety), (praziquantel AND pregnancy AND fetal outcomes), and (praziquantel AND pregnancy AND WHO).

### 2.3. Eligibility Criteria

Given the historical underrepresentation of pregnant women in clinical research, broad inclusion criteria were applied. Eligible sources included randomized controlled trials, prospective and retrospective cohort studies, case–control studies, case series, pharmacokinetic and pharmacodynamic studies, systematic reviews, meta-analyses, qualitative studies, and international guideline documents. Animal studies were included only when directly relevant to safety or pharmacological mechanisms.

Exclusion criteria used include the following:

- Non-pregnancy schistosomiasis studies unless providing mechanistic PK/PD data.
- Studies on parasitic infections other than schistosomiasis.
- Non-human studies are not directly relevant to fetal or maternal safety.
- Conference abstracts without extractable data.
- Non-English publications when translation was unavailable.

### 2.4. Study Selection and Screening Process

Titles and abstracts were screened for relevance. Full texts of potentially eligible studies were reviewed. Duplicate records were removed. A total of 60 studies met the inclusion criteria.

### 2.5. Data Extraction and Verification

**Figure 1:**
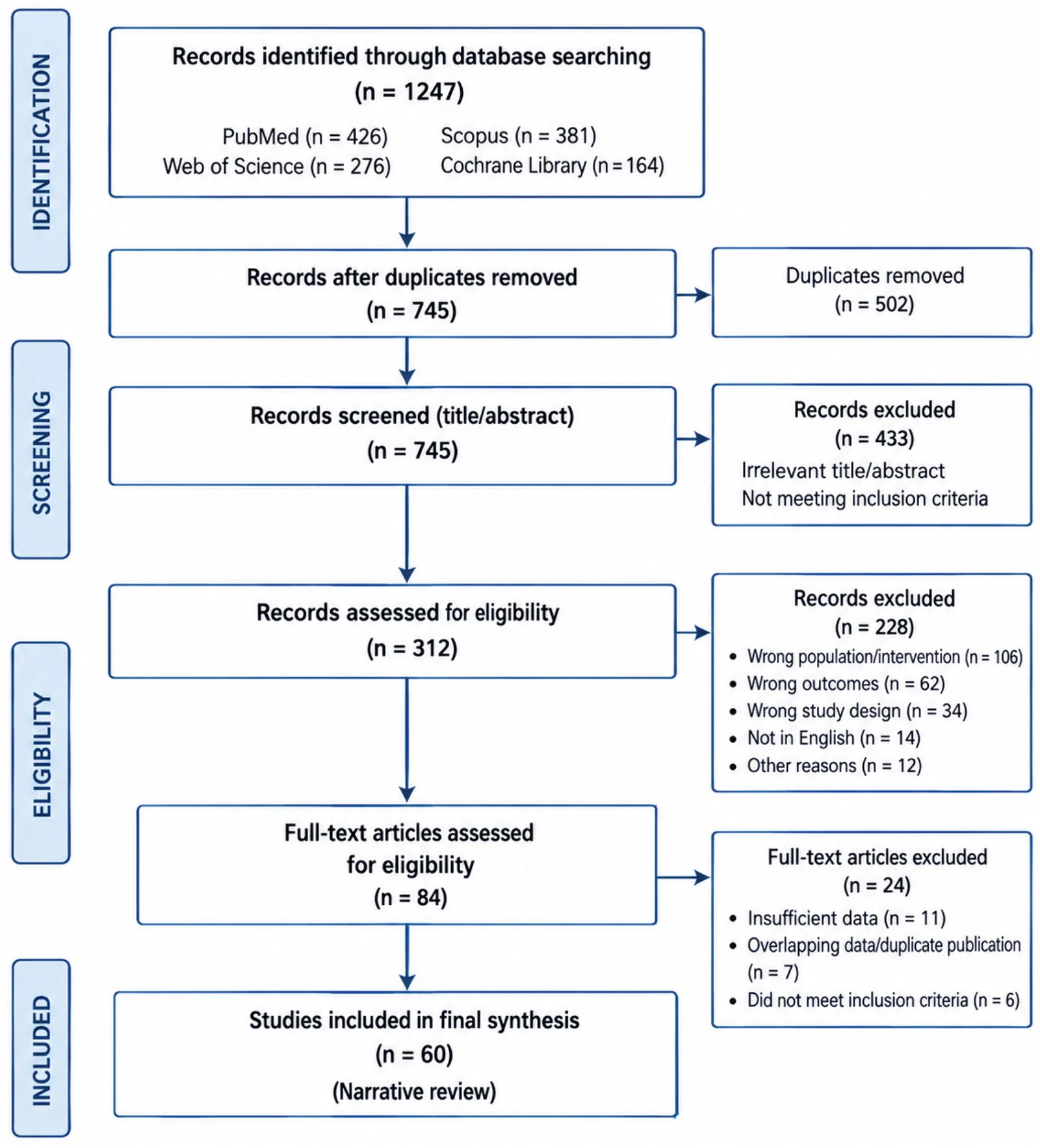
PRISMA flowchart of study selection for the narrative review.

A total of 1247 records were identified; 312 were screened; 84 full texts were reviewed; and 60 studies met inclusion criteria. Two reviewers independently screened records and resolved discrepancies by consensus. Data were extracted into structured matrices covering pharmacology, efficacy, safety, maternal outcomes, neonatal outcomes, and policy considerations. The study selection process is summarized in the PRISMA flowchart (Figure 1).

### 2.6. Data Synthesis

Data were extracted and organized into thematic domains: pharmacology, burden of disease, efficacy, safety, immunological effects, and global policy. Due to substantial heterogeneity in study populations, designs, and outcome measures, quantitative meta-analysis was not performed. Instead, the findings were synthesized qualitatively, with an emphasis on the consistency of evidence across study types and settings. The synthesis prioritized clarity, coherence, and alignment with SANRA recommendations.

### 2.7. Evidence Synthesis and Certainty Assessment

Evidence was synthesized across thematic domains. Certainty was judged based on study design hierarchy:

- High certainty: RCTs (second/third trimester).
- Moderate certainty: cohort studies (including first trimester).
- Low certainty: case series, pharmacovigilance reports, animal studies.

Safety outcomes were categorized into maternal, fetal, neonatal, and long-term offspring endpoints.

## 3. Results

### 3.1. Pharmacology of Praziquantel in Pregnancy

Praziquantel is a pyrazinoisoquinoline derivative that has served as the cornerstone of schistosomiasis treatment for over four decades. Understanding its pharmacological properties in the context of pregnancy is essential for assessing therapeutic efficacy, maternal tolerability, and fetal safety [26,27].

#### 3.1.1. Praziquantel: Mechanism of Action

Praziquantel exerts its antiparasitic effect by increasing the permeability of schistosome cell membranes to calcium ions, leading to sustained muscular contraction, paralysis, and subsequent tegumental disruption [5,6]. Tegumental damage exposes parasite antigens to the host immune system, facilitating immune-mediated parasite clearance [27]. This mechanism is effective against all major human schistosome species, including *Schistosoma mansoni*, *S. haematobium*, and *S. japonicum* [5].

Praziquantel (PZQ) targets the Sm.TRPMPZQ channel in schistosomes, activating it to induce a calcium ion influx, resulting in paralysis and surface damage of the parasites. The active enantiomer (R)-PZQ is more effective than (S)-PZQ, and its mechanism parallels pharmacological effects on muscle contraction and tegument disruption. This discovery may facilitate new treatments for schistosomiasis and address drug resistance by informing the design of similar drugs targeting other parasites. Experimental assays confirm that (R)-PZQ causes rapid paralysis and damage to adult schistosome worms [28].

Praziquantel’s selectivity for the schistosome-specific Sm. TRPMPZQ channel, which differs substantially from human TRP channels, provides a biological basis for its favorable safety profile. Common adverse reactions such as dizziness, nausea, and abdominal discomfort are self-limiting and correspond to transient peak plasma concentrations rather than off-target human channel activation.

#### 3.1.2. Praziquantel: Pharmacokinetics in Non-Pregnant Adults

In non-pregnant adults, praziquantel is rapidly absorbed following oral administration, with peak plasma concentrations occurring within 1–3 h [10]. The drug undergoes extensive first-pass hepatic metabolism, primarily via cytochrome P450 enzymes (notably CYP3A4), resulting in a short elimination half-life of approximately 1–2.5 h. Praziquantel exhibits high plasma protein binding (~80%) and is predominantly excreted as inactive metabolites via the kidneys [10].

#### 3.1.3. Praziquantel: Pregnancy-Related Pharmacokinetic Considerations

Physiological changes during pregnancy—including increased plasma volume, altered gastrointestinal motility, enhanced hepatic enzyme activity, and increased renal clearance—may theoretically influence drug disposition. However, available evidence indicates that these changes do not result in clinically meaningful alterations in praziquantel exposure [10].

Population pharmacokinetic analyses conducted among pregnant and lactating women infected with *S. japonicum* demonstrated that standard praziquantel dosing achieves therapeutic plasma concentrations comparable to those observed in non-pregnant adults [25]. No dose adjustment was required to maintain efficacy or safety, and interindividual variability was largely attributable to infection intensity rather than pregnancy status [25]. These findings are consistent with clinical trial data demonstrating preserved efficacy across gestational stages [8,9,24,29,30].

#### 3.1.4. Praziquantel: Placental Transfer and Fetal Exposure

Praziquantel is a small, lipophilic molecule and can cross the placental barrier. Nevertheless, several lines of evidence support the absence of clinically significant fetal risk. Animal studies have consistently demonstrated no teratogenic, embryotoxic, or mutagenic effects at therapeutic or supratherapeutic doses [11]. Human data accumulated over decades—including randomized controlled trials, cohort studies, and pharmacovigilance reports—have not identified any increase in congenital anomalies or adverse fetal outcomes attributable to praziquantel exposure [8,9,10,24,29,30,31].

Importantly, placental pathology in schistosomiasis reflects chronic inflammation from eggs, not drug toxicity [32]. Improved tissue maceration and histopathological techniques have further confirmed that placental involvement is a manifestation of untreated infection, reinforcing the rationale for treatment rather than avoidance [33].

#### 3.1.5. Praziquantel: Lactation

Praziquantel is excreted into breast milk in small quantities. Pharmacokinetic studies indicate that infant exposure through breastfeeding is minimal and transient [25]. WHO guidelines state that breastfeeding is not a contraindication to praziquantel therapy, and no data exists on effects in breastfed infants or milk production following maternal praziquantel treatment [12,13,14].

#### 3.1.6. Praziquantel: Drug–Drug Interactions

Praziquantel has 14 major drug interactions (highly clinically significant; avoid combinations; the risk of interaction outweighs the benefit), most of which involve agents that strongly alter CYP450 metabolism, leading to clinically significant changes in praziquantel exposure. The major interactions include potent CYP3A inducers such as rifampin, carbamazepine, phenytoin, phenobarbital, rifabutin, and St. John’s wort, all of which can markedly reduce praziquantel plasma levels and compromise efficacy. Several CYP3A inhibitors, including ritonavir (as part of Paxlovid), may substantially increase praziquantel concentrations, raising the risk of adverse effects. These interactions are classified as highly clinically significant, and combinations should generally be avoided [34].

Praziquantel has one notable food interaction, primarily involving grapefruit products. Food intake increases praziquantel absorption, so the drug should be taken with or immediately after a meal to maximize bioavailability. However, grapefruit and grapefruit juice should be avoided, as they can significantly raise praziquantel blood levels and increase the likelihood of side effects such as dizziness, abdominal discomfort, and nausea. Patients who regularly consume grapefruit should consult their clinician before making any dietary changes [35,36].

#### 3.1.7. Praziquantel: Pharmacology Summary

Available pharmacological and pharmacokinetic data support the biological plausibility of praziquantel’s safety and efficacy during pregnancy. Pregnancy-related physiological changes do not necessitate a dose modification, and placental transfer has not been associated with fetal harm. These findings provide a robust pharmacological foundation for WHO recommendations (Recommendation 4) supporting the use of praziquantel in pregnancy and lactation. WHO Recommendation 4 states that: WHO recommends that health facilities (In endemic communities with prevalence of *Schistosoma* spp. infection ≥ 10%) provide access to treatment with praziquantel to control morbidity due to schistosomiasis in all infected individuals regardless of age, including infected pregnant women (excluding the first trimester), lactating women and pre-SAC aged <2 years. It is considered a strong recommendation by the WHO, and the certainty of the evidence is moderate [14].

### 3.2. Burden of Schistosomiasis in Pregnancy

Schistosomiasis in pregnancy represents a substantial yet historically underrecognized component of the global disease burden. The intersection of chronic parasitic infection and pregnancy-related physiological demands has important implications for maternal health, fetal development, and long-term child outcomes.

#### 3.2.1. Epidemiology

An estimated 10–12 million pregnant women worldwide are infected with schistosomes each year, with the highest prevalence observed in sub-Saharan Africa, Southeast Asia, and parts of South America and the Middle East [1,2,3,4]. Exposure persists during pregnancy due to continued contact with infested freshwater through domestic, agricultural, and occupational activities.

Urogenital schistosomiasis caused by *S. haematobium* is particularly prevalent among women of reproductive age, while intestinal and hepatosplenic disease due to *S. mansoni* and *S. japonicum* remain common in endemic regions [2,17].

#### 3.2.2. Maternal Morbidity

##### Anemia

Anemia is the most consistently documented maternal complication of schistosomiasis in pregnancy [37]. Chronic blood loss, systemic inflammation, and impaired iron metabolism contribute to iron deficiency anemia and anemia of inflammation in all populations [38,39]. A recent systematic review and meta-analysis confirmed a significant association between schistosomiasis and maternal anemia among pregnant women [37]. Anemia during pregnancy is a major risk factor for preterm birth, low birth weight, postpartum hemorrhage, and maternal mortality [40,41].

##### Urogenital and Hepatosplenic Morbidity

Urogenital schistosomiasis, in all patients, causes hematuria, genital lesions, dysuria, and chronic pelvic inflammation, which may complicate pregnancy and increase susceptibility to secondary infections, including HIV [42,43]. Chronic intestinal schistosomiasis may progress to periportal fibrosis, splenomegaly, and portal hypertension, posing additional risks during pregnancy in advanced cases [17,20,44].

##### Inflammation and Immune Dysregulation

Pregnancy is characterized by dynamic immunological modulation. Superimposed chronic schistosomiasis may exacerbate systemic inflammation, eosinophilia, and immune dysregulation, potentially impairing placental function and maternal nutritional status [18,19,23,45,46,47].

#### 3.2.3. Fetal and Neonatal Consequences

Although schistosomes do not directly infect the fetus, maternal infection can adversely affect fetal development through indirect mechanisms (Section 3.1.4).

##### Low Birth Weight and Growth Restriction

Multiple prospective cohort studies from Gabon and southern Africa have demonstrated an association between maternal schistosomiasis—particularly urogenital infection—and reduced birth weight [20,21,48,49]. Polyparasitic infection further amplifies this risk [21]. Importantly, subsequent analyses have emphasized that these adverse outcomes are attributable to infection-related morbidity rather than praziquantel treatment itself [49].

##### Neonatal and Early Childhood Outcomes

Maternal schistosomiasis has been associated with impaired neonatal iron status and altered immune development in early childhood [22,23,50]. These findings underscore the potential intergenerational consequences of untreated infection during pregnancy.

#### 3.2.4. Socioeconomic and Structural Determinants

Women in endemic settings frequently face barriers to diagnosis and treatment, including limited access to antenatal care, inadequate diagnostic infrastructure, gender-based inequities, and exclusion from MDA campaigns due to pregnancy status [15,44,51,52]. These factors contribute to persistent, untreated infection and chronic morbidity.

#### 3.2.5. Public Health Implications

The burden of schistosomiasis in pregnancy extends beyond individual health effects, contributing to adverse birth outcomes, impaired child development, and sustained community transmission [53,54]. Addressing schistosomiasis during pregnancy represents a critical opportunity to improve maternal health, enhance neonatal outcomes, and advance global elimination goals [22,55].

### 3.3. Efficacy of Praziquantel in Pregnancy

Praziquantel has long been established as the most effective treatment for schistosomiasis in non-pregnant populations. Evaluating its efficacy during pregnancy requires consideration of parasitological outcomes, maternal clinical benefits, and potential downstream effects on fetal and child health. Evidence from randomized controlled trials (RCTs), observational cohorts, and post-treatment consistently demonstrates that praziquantel retains high efficacy during pregnancy, as shown below:

#### 3.3.1. Evidence from Randomized Controlled Trials

RCTs provide the strongest evidence for praziquantel efficacy in pregnancy. To date, several well-designed trials conducted across different epidemiological settings have evaluated praziquantel against major schistosome species.

##### *Schistosoma* *mansoni*

In a randomized, placebo-controlled trial conducted in Uganda, pregnant women infected with *S. mansoni* received praziquantel at a standard dose of 40 mg/kg during the second or third trimester. Treatment resulted in significant reductions in egg counts and cure rates comparable to those observed in non-pregnant adults [23,50]. Importantly, pregnancy-related physiological changes did not compromise parasitological efficacy.

##### *Schistosoma* *japonicum*

One landmark RCT conducted in the Philippines evaluated praziquantel treatment in pregnant women infected with *S. japonicum.* Olveda et al. demonstrated high cure rates and substantial egg reduction following treatment with 60 mg/kg praziquantel administered in divided doses [9]. Maternal treatment was also associated with improvements in hemoglobin and iron indices, highlighting potential benefits beyond parasite clearance [9].

##### *Schistosoma* *haematobium*

The most recent evidence comes from the freeBILy Gabon randomized, single-blinded controlled trial, which evaluated the efficacy of praziquantel in pregnant women infected with *S. haematobium.* Treatment resulted in high cure rates, marked reductions in egg excretion, and significant improvement in urogenital morbidity [24]. Efficacy was consistent across the second gestational trimester in this study, further supporting the use of praziquantel in the 2nd trimester of pregnancy.

The key randomized controlled trials evaluating praziquantel use during pregnancy across *S. mansoni*, *S. japonicum*, and *S. haematobium* demonstrate consistently high cure rates and no adverse pregnancy outcomes. These findings are summarized in Table 1, which provides a comparative overview of randomized controlled trials evaluating the use of praziquantel during pregnancy. It summarizes study locations, schistosome species, dosing regimens, gestational timing, and key maternal and fetal outcomes. Collectively, these trials demonstrate high therapeutic efficacy and no increase in adverse pregnancy events.

#### 3.3.2. Evidence from Observational Studies

Observational studies conducted in endemic regions provide complementary real-world evidence supporting the efficacy of praziquantel in pregnancy. Cohort studies from Sudan have consistently reported high parasitological cure rates following treatment [29,30]. These findings are particularly valuable for assessing treatment effectiveness in first-trimester exposures and in routine clinical settings where RCT data are limited.

#### 3.3.3. Impact on Maternal Morbidity

Beyond parasitological outcomes, praziquantel treatment during pregnancy confers measurable maternal-health benefits as detailed below.

##### Anemia and Nutritional Status

Multiple studies have demonstrated improvements in maternal hemoglobin levels following praziquantel treatment, particularly among women with moderate-to-heavy infections with *S. haematobium* [24]. Given the strong association between schistosomiasis and maternal anemia [37], these improvements are clinically meaningful and may reduce obstetric risk. On the other hand, one study (*Schistosoma mansoni* infection) found no overall effect of praziquantel on maternal anemia [8].

##### Urogenital Morbidity

In *S. haematobium* infection, for pregnant and non-pregnant patients, praziquantel treatment reduces hematuria, genital lesions, and chronic urogenital inflammation [24,43]. These effects may lower susceptibility to secondary infections and improve quality of life during pregnancy.

#### 3.3.4. Effects on Fetal and Neonatal Outcomes

RCTs and cohort studies consistently demonstrate that praziquantel treatment does not adversely affect fetal growth or neonatal outcomes. Trials from Uganda and the Philippines reported no reduction in birth weight, abortion, fetal death in utero, and congenital anomalies. Among treated women compared with controls [9] and healthier early immune development [22]. Some studies observed modest improvements in neonatal iron stores among infants born to treated mothers, likely mediated by improved maternal iron status [55].

#### 3.3.5. Immunological Effects and Offspring Outcomes

Increasing attention has been directed toward the immunological consequences of maternal schistosomiasis and praziquantel treatment.

RCTs evaluating cytokine responses have shown that praziquantel treatment during pregnancy modulates maternal immune responses to schistosome antigens without adverse clinical consequences [50]. This effect was also observed in nonpregnant women [56]. Long-term follow-up studies of offspring demonstrated that maternal infection and treatment influence immune responsiveness in early childhood, but do not increase susceptibility to schistosomiasis or other infections [23,48].

Experimental and observational evidence suggests that reducing chronic antigenic exposure during gestation may have favorable effects on immune programming in offspring [32]. These findings further support treatment during pregnancy as a strategy to mitigate intergenerational effects of schistosomiasis.

#### 3.3.6. Summary of Efficacy Evidence

Across diverse settings and schistosome species, praziquantel demonstrates consistently high efficacy during pregnancy. Cure rates, egg reduction, and clinical benefits are comparable to those observed in non-pregnant adults, with additional potential benefits for maternal anemia and early childhood health. Pregnancy does not appear to diminish therapeutic response, reinforcing the appropriateness of standard dosing regimens.

### 3.4. Safety of Praziquantel in Pregnancy

The safety of praziquantel during pregnancy has been evaluated through multiple complementary evidence streams, including RCTs, observational cohorts, case series, pharmacokinetic studies, and global pharmacovigilance data. Together, these data provide one of the most robust safety profiles available for any antiparasitic drug used in pregnancy.

#### 3.4.1. Evidence from Randomized Controlled Trials

RCTs conducted in Uganda (2nd or 3rd trimester), the Philippines (at 12–16 weeks gestation), and Gabon (2nd trimester) consistently demonstrate that praziquantel treatment during pregnancy is not associated with adverse maternal or fetal outcomes.

Across these trials, no increases were observed in miscarriage, stillbirth, congenital anomalies, preterm delivery, or neonatal mortality among treated women compared with placebo or untreated controls [8,9,24,55]. Maternal adverse events—such as dizziness, nausea, headache, and abdominal discomfort—were mild, transient, and consistent with the known tolerability profile of praziquantel in non-pregnant populations.

#### 3.4.2. Observational Cohort Studies

Observational data are particularly valuable for assessing first-trimester exposure, which is often excluded from RCTs. Retrospective and prospective cohorts from Sudan involving women inadvertently treated during early pregnancy reported no increase in spontaneous abortion, stillbirth, or congenital anomalies compared with unexposed pregnancies (the study addressed safety across all trimesters) [29,30].

Additional meta-analysis studies, similarly, found no association between praziquantel exposure and adverse pregnancy outcomes, even in settings with high infection intensity and limited healthcare infrastructure [10,31].

Evidence from observational cohorts and case series further supports the safety of praziquantel during pregnancy, including first-trimester exposures. When comparing praziquantel, albendazole, and mebendazole, praziquantel shows the most reassuring safety profile across all trimesters, with no evidence of reproductive or fetal toxicity at therapeutic doses and animal data supporting normal maternal and fetal outcomes. In contrast, albendazole is contraindicated in the first trimester due to potential embryotoxicity at very high doses but is generally considered acceptable in the second and third trimesters when used at standard clinical exposures. Mebendazole is of greatest concern: it has shown teratogenic effects in rats, is contraindicated during pregnancy—especially in the first trimester—and although a “No-Observed-Adverse-Effect Level” (NOAEL) has been identified in animal studies, its overall safety margin is narrower than that of praziquantel or albendazole later in pregnancy. Table 2 compiles evidence from observational studies assessing the safety of praziquantel in real-world settings. These studies include first-trimester exposures and high-dose accidental treatments, all of which consistently report no teratogenic or obstetric risks.

#### 3.4.3. High-Dose Exposure and Case Series

Because several early case reports and small case series documenting praziquantel use during pregnancy were published in non-indexed Chinese parasitology journals, they were not retrievable through PubMed, Web of Science, or Saudi Digital Library (SDL). These three reports, however, are cited in WHO technical documents and global schistosomiasis reviews [57]. They include a 4-case series of women treated at 3–4 months’ gestation [58], and two individual case reports describing treatment at 27 weeks [59] and 8 months’ gestation [60]. All women were cured and delivered healthy infants, with no congenital anomalies reported.

#### 3.4.4. Pharmacovigilance and Long-Term Safety Data

Praziquantel has been used globally since the late 1970s, with hundreds of millions of doses administered. Post-marketing surveillance and WHO safety reviews [61] have not identified any teratogenic signal or pattern of adverse reproductive outcomes attributable to praziquantel [10,11]. The Olveda study is the largest and most rigorous trial evaluating praziquantel in pregnant women [9]. Although technically an RCT, the Olveda study was conducted after praziquantel approval and functions as a Phase IV safety study. It provides strong evidence of maternal and fetal safety. Also, clinical results from the freeBILy-Gabon trial, a randomized, single-blinded, controlled trial, evaluated the safety and efficacy of praziquantel in pregnant women infected with *Schistosoma haematobium*. It was also a post-approval trial that contributed real-world pregnancy safety data [24].

Evidence is broadly consistent, though gaps remain in first trimester and long-term offspring outcomes.

#### 3.4.5. First-Trimester Exposure

RCT data are limited for first-trimester treatment; available observational evidence does not indicate an increased risk of embryotoxicity or teratogenicity [29,30]. In endemic communities with prevalence of *Schistosoma* spp. infection ≥ 10%, WHO recommends annual preventive chemotherapy with a single dose of praziquantel at ≥75% treatment coverage in all age groups from 2 years old, including adults, pregnant women after the first trimester and lactating women, to control schistosomiasis morbidity and advance towards eliminating the disease as a public health problem [12,13,14].

#### 3.4.6. Lactation Safety

Praziquantel appears in breast milk only at low concentrations, and available data do not identify any known effects on breastfed infants or milk production [3]. WHO guidelines explicitly state that breastfeeding is not a contraindication to treatment, supporting praziquantel use in the postpartum period, especially in endemic communities with prevalence of *Schistosoma* spp. infection ≥ 10% [12,13,14]. When prescribing Praziquantel, clinicians should weigh the benefits of breastfeeding against the mother’s clinical need for treatment and any theoretical risks to the infant associated with the medication or the underlying maternal condition.

#### 3.4.7. Summary of Safety Evidence

The drug’s long history of use, combined with high-quality RCTs and extensive observational data, provides a level of reassurance rarely available for medications used in pregnancy. Safety evidence is strong for the second and third trimesters and moderate for the first trimester.

### 3.5. WHO Recommendations and the Global Policy Landscape

#### 3.5.1. Evolution of WHO Guidance

The World Health Organization (WHO) plays a central role in defining global policy for schistosomiasis control. During the early years following the introduction of praziquantel, WHO adopted a precautionary approach regarding its use in pregnancy, largely due to limited human data rather than evidence of harm. Consequently, pregnant women were routinely excluded from mass drug administration (MDA) campaigns throughout the 1980s and 1990s.

A major policy shift occurred in 2002, when the WHO convened an expert consultation to review animal toxicology studies, post-marketing surveillance data, and emerging human observational evidence. The consultation concluded that praziquantel posed no demonstrable risk to the fetus and that the morbidity associated with untreated schistosomiasis outweighed any theoretical concerns (specifically in the 2nd and 3rd trimesters). In endemic communities with prevalence of *Schistosoma* spp. infection ≥ 10%, WHO recommends annual preventive chemotherapy with a single dose of praziquantel at ≥75% treatment coverage in all age groups from 2 years old, including adults, pregnant women after the first trimester, and lactating women, to control schistosomiasis morbidity and advance towards eliminating the disease as a public health problem [12,13,14].

This recommendation was reaffirmed in later technical reports and incorporated into WHO guidelines on preventive chemotherapy and schistosomiasis control [13,14,62]. The most recent WHO guideline on schistosomiasis control and elimination continues to endorse the use of praziquantel for pregnant women after the first trimester and lactating women and emphasizes the inclusion of these women in treatment strategies [14].

#### 3.5.2. Rationale for Treating Pregnant Women

WHO’s recommendations are grounded in four principal considerations:

- Robust safety evidence, including randomized controlled trials, large observational cohorts, and decades of pharmacovigilance, demonstrates no increased risk of adverse pregnancy outcomes [8,9,10,12,13,14,24,29,30,31].
- In all populations, including pregnant women with high disease burden, schistosomiasis contributes to maternal anemia, chronic inflammation, urogenital pathology, and impaired nutritional status [17,18,19,43].
- Public health impact, whereby treating pregnant women reduces individual morbidity and may contribute to decreased community transmission [2,63].
- Ethical considerations, as exclusion of pregnant women from treatment perpetuates gender inequities and denies evidence-based care [15,42].

#### 3.5.3. Persistent Policy–Practice Gaps

Despite clear WHO guidance, many national schistosomiasis control programs continue to exclude pregnant women from MDA campaigns. This policy–practice gap is driven by multiple factors:

- Outdated national guidelines that have not been revised to reflect WHO recommendations.
- Healthcare worker misconceptions regarding drug safety in pregnancy, often reinforced by conservative drug labeling [16,64].
- Operational challenges, including difficulties identifying pregnant women during MDA campaigns and concerns about liability.
- Sociocultural factors, such as community fears regarding medication use during pregnancy.

Qualitative research from Zanzibar and other endemic settings demonstrates that pregnant women are often willing to receive praziquantel when appropriately informed, indicating that implementation barriers are modifiable [15].

Despite strong WHO recommendations, many national programs continue to exclude pregnant women from mass drug administration campaigns. Table 3 contrasts WHO recommendations with common national practices regarding praziquantel use in pregnancy and lactation. It highlights persistent policy–practice gaps that contribute to untreated maternal infections and hinder schistosomiasis elimination efforts.

#### 3.5.4. Implications for Elimination Strategies

WHO’s current elimination framework emphasizes a life-course approach to schistosomiasis control, recognizing the importance of protecting vulnerable populations, including pregnant women and young children [62,63]. Excluding pregnant women from treatment undermines elimination goals by allowing persistent reservoirs of infection and preventable morbidity.

## 4. Discussion

This review synthesizes four decades of evidence on the use of praziquantel during pregnancy, integrating randomized trials, observational cohort studies, pharmacokinetic studies, case series, and WHO policy documents. Collectively, the findings demonstrate that praziquantel is effective and generally safe for pregnant women, although the strength of evidence varies by species, trimester, and study design. A critical appraisal of these differences is essential for accurate interpretation and for guiding clinical and public health decision-making.

### 4.1. Strength of Evidence Across Study Designs

Randomized controlled trials provide the highest-certainty evidence and consistently show that praziquantel is effective and well tolerated when administered during the second and third trimesters. These trials, conducted in Uganda, the Philippines, and Gabon, demonstrate high cure rates, substantial egg reduction, and no increase in miscarriage, stillbirth, congenital anomalies, preterm birth, or neonatal mortality. Maternal adverse events were mild and transient, aligning with the known tolerability profile in non-pregnant adults.

Observational studies offer complementary insights, particularly for first-trimester exposures, which are rarely included in RCTs. Cohort studies from Sudan, including accidental early-pregnancy exposures, have not identified teratogenic signals or increased obstetric risk. Although observational designs are inherently more susceptible to confounding, the consistency of findings across diverse settings strengthens confidence in praziquantel’s safety profile.

Pharmacokinetic studies further support the biological plausibility of safety. Pregnancy-related physiological changes do not significantly alter praziquantel exposure, and standard dosing achieves therapeutic concentrations comparable to those in non-pregnant adults. These findings reinforce the appropriateness of current dosing recommendations.

Praziquantel’s selectivity for the schistosome-specific Sm.TRPMPZQ channel, which differs substantially from human TRP channels, provides a mechanistic explanation for its favorable safety profile. Common adverse reactions (e.g., dizziness, nausea) correspond to transient peak plasma levels rather than off-target human channel activation.

### 4.2. Species-Specific Considerations

Evidence strength varies across schistosome species.

- *S. mansoni*: Supported by multiple RCTs and observational studies, providing high-certainty evidence for efficacy and safety.
- *S. japonicum*: Two well-designed RCTs demonstrate strong efficacy and maternal hematologic benefits, though fewer studies exist compared with S. mansoni.
- *S. haematobium*: Evidence includes the recent freeBILy RCT and several observational studies, but fewer trials have evaluated early-pregnancy exposure.
- Despite these differences, no species-specific safety concerns have been identified.

### 4.3. Trimester Specific Evidence

The evidence base is strongest for 2nd- and 3rd-trimester treatment, where RCTs consistently demonstrate safety and efficacy. First-trimester evidence is more limited and derives primarily from observational cohorts and case series. These studies do not indicate an increased risk of embryotoxicity or teratogenicity, but the absence of first-trimester RCTs warrants cautious interpretation. Future research should prioritize early-pregnancy exposures to strengthen trimester-specific certainty.

### 4.4. Maternal and Neonatal Benefits of Treatment

Beyond parasitological cure, praziquantel treatment confers important maternal-health benefits. Improvements in hemoglobin and iron indices have been documented in moderate-to-heavy infections, and reductions in hematuria and genital lesions have been observed, improving urogenital health and quality of life. These maternal benefits may translate into improved neonatal iron status and healthier early immune development, although long-term offspring data remain limited.

### 4.5. WHO Guidance and Policy–Practice Gaps

WHO has considered praziquantel treatment during pregnancy since 2002, based on accumulated safety evidence and the substantial morbidity associated with untreated infection. However, the 2022 WHO guideline recommends praziquantel treatment during pregnancy but explicitly recommends treatment after the first trimester (recommend treatment only during 2nd and 3rd trimesters), reflecting a cautious approach to early-pregnancy exposure.

Despite WHO guidance, many national programs continue to exclude pregnant women from MDA campaigns. This policy–practice gap is driven by outdated national guidelines, healthcare worker misconceptions, operational challenges in identifying pregnancy during MDA, liability concerns, and sociocultural hesitancy. These barriers result in millions of untreated infections annually and undermine schistosomiasis elimination efforts.

A structured comparison of treated versus untreated schistosomiasis shows clear advantages of treatment across parasitological, hematologic, urogenital, and neonatal domains. Untreated infection is associated with anemia, chronic inflammation, urogenital morbidity, low birth weight, and impaired neonatal iron status, whereas treated women experience improved hemoglobin, reduced hematuria, and no increase in adverse pregnancy outcomes, as shown in Table 4 below.

### 4.6. Implications for Clinical Practice and Public Health

Integrating praziquantel into antenatal care could substantially reduce maternal morbidity, improve neonatal outcomes, and contribute to community-level transmission reduction. Given the strong evidence supporting treatment in late pregnancy and the reassuring observational data in early pregnancy, clinicians should feel confident in offering praziquantel when schistosomiasis is diagnosed or strongly suspected.

### 4.7. Limitations of the Evidence Base

Several limitations warrant consideration.

- First-trimester RCT data are lacking.
- Long-term offspring follow-up is limited.
- Diagnostic methods and outcome measures vary across studies.
- Rare adverse events cannot be fully excluded.
- Evidence strength differs across schistosome species.
- Some observational studies rely on retrospective data with potential misclassification.
- These limitations highlight the need for future research, particularly research regarding first-trimester exposure and long-term child outcomes.

### 4.8. Persistent Policy–Practice Gaps

When comparing praziquantel policy to real-world implementation, a persistent gap remains between WHO recommendations and national practice. Many schistosomiasis control programs still exclude pregnant women from mass drug administration, largely due to outdated national guidelines, misconceptions among healthcare workers about drug safety in pregnancy, and operational concerns related to identifying pregnant women and managing liability during campaigns. Sociocultural fears surrounding medication use in pregnancy further reinforce these barriers, collectively contributing to continued under-treatment of pregnant women despite strong evidence supporting praziquantel’s safety and effectiveness.

Considering the disease-wide spectrum from mild egg excretion to severe hepatosplenic disease (and integration with WHO prevalence-based strategies to guide community-level implementation) the authors suggest a stratified approach to manage schistosomiases during pregnancy that may improve acceptability:

- Mild infections: monitored deferral until the end of the 1st trimester, or postpartum when clinically appropriate.
- Moderate/severe infections or anemia/urogenital symptoms: active treatment during pregnancy, with repeat dosing considered in high-intensity infections.

## 5. Conclusions

Praziquantel is effective and generally safe during pregnancy, with the strongest evidence in the second and third trimesters. Treatment improves maternal anemia and may benefit neonatal iron status and immune development. Evidence gaps remain regarding first-trimester exposures and long-term outcomes. Strengthening national policies and integrating praziquantel into antenatal care could substantially reduce maternal morbidity.

## Figures and Tables

**Table 1:** Randomized controlled trials of praziquantel use during pregnancy.

Study	Country	Species	Sample Size	Design	Trimester	Dose	Comparator	Cure Rate	Egg Reduction	Maternal AEs	Pregnancy Outcomes
Tweyongyere et al., 2008 [22]	Uganda	*S. mansoni*	387 (subset)	RCT (immunology)	2nd–3rd	40 mg/kg	Placebo	88%	90%	Mild, transient	No difference in miscarriage, stillbirth, LBW
Olveda et al., 2016 [9]	Philippines	*S. japonicum*	370	Double-blind RCT	2nd–3rd	60 mg/kg	Placebo	92%	95%	Mild	Improved maternal Hb; no adverse fetal outcomes
Gerstenberg et al., 2024 (freeBILy) [24]	Gabon	*S. haematobium*	334	RCT	2nd trimester	40 mg/kg	Placebo	85%	89%	Mild	Reduced hematuria; no adverse pregnancy outcomes

**Table 2:** Observational Studies and Case Series on Praziquantel Safety in Pregnancy.

Study	Country	Species	Sample Size	Trimester	Design	Cure Rate	Egg Reduction	Maternal Outcomes	Pregnancy Outcomes
Adam et al., 2005 [29]	Sudan	*S. mansoni*	25	1st–3rd	Cohort	Not mentioned	Not mentioned	Treatment success not mentioned, no adverse effects	No stillbirths, pre-term labor or maternal deaths
Adam et al., 2004 [30]	Sudan	Not mentioned	88	1st–3rd	Cohort	Not mentioned	Not mentioned	Treatment success not mentioned	No increase in abortions or preterm deliveries

**Table 3:** WHO Recommendations versus National Practice.

Organization	Year	Recommendation	Trimester Specificity	Notes
WHO [3]	2002	WHO held an Informal Consultation to assess available data	Study use after the 1st trimester	Reaffirmed in later guidelines
WHO	2022	Praziquantel may be given during pregnancy	recommended in the 2nd & 3rd trimesters	Emphasizes safety but cautious wording
CDC [65]	2024	Treatment recommended when benefits outweigh potential risks	No trimester restriction	Based on accumulated safety data
National Programs (various)	2010–2025	Mixed policies	Often exclude pregnancy	Outdated guidelines and provider hesitancy

**Table 4:** Comparison of Treated vs. Untreated Schistosomiasis in Pregnancy.

Outcome Domain	Untreated Schistosomiasis	Praziquantel-Treated Pregnancy
Parasitological	Persistent egg excretion; chronic infection and hinders schistosomiasis elimination efforts.	High cure rate; marked egg reduction
Maternal anemia	Increased risk of moderate–severe anemia	Improved hemoglobin and iron indices
Urogenital morbidity	Hematuria, genital lesions, chronic inflammation	Resolution of hematuria; reduced lesions
Pregnancy outcomes	Higher risk of LBW, IUGR, preterm birth	No increase in miscarriage, stillbirth, LBW
Neonatal iron status	Reduced neonatal ferritin and Hb	Improved neonatal iron indices (Philippines RCT)
Immune development	Altered neonatal immune programming	More regulated cytokine responses; no adverse effects
Long-term child outcomes	Potential impaired early immunity	No increased infection susceptibility

## Data Availability

No new data were created or analyzed in this study.

## Abbreviations

The following abbreviations are used in this manuscript:

Abbreviation	Meaning
WHO	World Health Organization
MDA	Mass Drug Administration
PZQ	Praziquantel
RCT	Randomized Controlled Trial
FDA	Food and Drug Administration
PK	Pharmacokinetics
*S. mansoni*	*Schistosoma mansoni*
*S. haematobium*	*Schistosoma haematobium*
*S. japonicum*	*Schistosoma japonicum*
TRP	Transient Receptor Potential (ion channel family)
GST	Glutathione-S-Transferase (vaccine candidate antigen)
HIV	Human Immunodeficiency Virus
STH	Soil-Transmitted Helminths
IRB	Institutional Review Board

## References

[1] WHO (2006). Preventive Chemotherapy in Human Helminthiasis: A Manual for Health Professionals and Programme Managers.

[2] Freer J.B., Bourke C.D., Durhuus G.H., Kjetland E.F., Prendergast A.J. (2018). Schistosomiasis in the First 1000 Days. Lancet Infect. Dis..

[3] WHO Expert Committee (2002). Report of the WHO Informal Consultation on the Use of Praziquantel During Pregnancy/Lactation and Albendazole/Mebendazole in Children Under 24 Months: Geneva, 8–9 April 2002.

[4] Chitsulo L., Engels D., Montresor A., Savioli L. (2000). The Global Status of Schistosomiasis and Its Control. Acta Trop..

[5] Doenhoff M.J., Cioli D., Utzinger J. (2008). Praziquantel: Mechanisms of Action, Resistance and New Derivatives for Schistosomiasis. Curr. Opin. Infect. Dis..

[6] Cioli D., Pica-Mattoccia L. (2003). Praziquantel. Parasitol. Res..

[7] Ross A.G.P., Bartley P.B., Sleigh A.C., Olds G.R., Li Y., Williams G.M., McManus D.P. (2002). Schistosomiasis. N. Engl. J. Med..

[8] Ndibazza J., Muhangi L., Akishule D., Kiggundu M., Ameke C., Oweka J., Kizindo R., Duong T., Kleinschmidt I., Muwanga M. (2010). Effects of Deworming during Pregnancy on Maternal and Perinatal Outcomes in Entebbe, Uganda: A Randomized Controlled Trial. Clin. Infect. Dis..

[9] Olveda R.M., Acosta L.P., Tallo V., Baltazar P.I., Lesiguez J.L.S., Estanislao G.G., Ayaso E.B., Monterde D.B.S., Ida A., Watson N. (2016). A Randomized Double Blind Placebo Controlled Trial Assessing the Efficacy and Safety of Praziquantel for the Treatment of Human Schistosomiasis during Pregnancy. Lancet Infect. Dis..

[10] Friedman J.F., Olveda R.M., Mirochnick M.H., Bustinduy A.L., Elliott A.M. (2018). Praziquantel for the Treatment of Schistosomiasis during Human Pregnancy. Bull. World Health Organ..

[11] U.S. Food and Drug Administration, Center for Drug Evaluation and Research (2023). Biltricide (Praziquantel) Tablets, for Oral Use. Prescribing Information.

[12] WHO Schistosomiasis: Progress Report 2001–2011, Strategic Plan 2012–2020. https://www.who.int/publications/i/item/978941503174.

[13] WHO (2020). Ending the Neglect to Attain the Sustainable Development Goals: A Road Map for Neglected Tropical Diseases 2021–2030.

[14] World Health Organization (2022). WHO Guideline on Control and Elimination of Human Schistosomiasis.

[15] De Rosa E., Person B., Knopp S., Muhsin J., Lyimo J.H., Kabole F., Rollinson D. (2022). A Descriptive Qualitative Case Study of the Experiences, Perceptions and Attitudes of Pregnant Women on Unguja Island, Zanzibar, towards Antischistosomal Treatment. Acta Trop..

[16] Adegnika A.A., Ramharter M., Agnandji S.T., Ateba Ngoa U., Issifou S., Yazdanbahksh M., Kremsner P.G. (2010). Epidemiology of Parasitic Co-Infections during Pregnancy in Lambaréné, Gabon. Trop. Med. Int. Health.

[17] King C.H., Dangerfield-Cha M. (2008). The Unacknowledged Impact of Chronic Schistosomiasis. Chronic Illn..

[18] Gryseels B., Polman K., Clerinx J., Kestens L. (2006). Human Schistosomiasis. Lancet.

[19] Friedman J.F., Kanzaria H.K., McGarvey S.T. (2005). Human Schistosomiasis and Anemia: The Relationship and Potential Mechanisms. Trends Parasitol..

[20] Mombo-Ngoma G., Honkpehedji J., Basra A., Mackanga J.R., Zoleko R.M., Zinsou J., Agobe J.C.D., Lell B., Matsiegui P.B., Gonzales R. (2017). Urogenital Schistosomiasis during Pregnancy Is Associated with Low Birth Weight Delivery: Analysis of a Prospective Cohort of Pregnant Women and Their Offspring in Gabon. Int. J. Parasitol..

[21] Honkpéhèdji Y.J., Adegbite B.R., Zinsou J.F., Dejon-Agobé J.C., Edoa J.R., Zoleko Manego R., McCall M., Mbong Ngwese M., Lotola Mougeni F., Mombo-Ngoma G. (2021). Association of Low Birth Weight and Polyparasitic Infection during Pregnancy in Lambaréné, Gabon. Trop. Med. Int. Health.

[22] Tweyongyere R., Mawa P.A., Ngom-wegi S., Ndibazza J., Duong T., Vennervald B.J., Dunne D.W., Katunguka-Rwakishaya E., Elliott A.M. (2008). Effect of Praziquantel Treatment during Pregnancy on Cytokine Responses to Schistosome Antigens: Results of a Randomized, Placebo-Controlled Trial. J. Infect. Dis..

[23] Tweyongyere R., Naniima P., Mawa P.A., Jones F.M., Webb E.L., Cose S., Dunne D.W., Elliott A.M. (2013). Effect of Maternal *Schistosoma Mansoni* Infection and Praziquantel Treatment During Pregnancy on *Schistosoma Mansoni* Infection and Immune Responsiveness among Offspring at Age Five Years. PLoS Negl. Trop. Dis..

[24] Gerstenberg J., Honkpehedji Y.J., Dejon-Agobe J.C., Mahmoudou S., Recker M., Mba R.B., Maloum M.N., Lontchi R.L., Moure P.A.N., Meulah B. (2024). Safety and Efficacy of Praziquantel in Pregnant Women Infected with *Schistosoma haematobium* in Lambaréné, Gabon—Clinical Results from the Randomized, Single-Blinded, Controlled FreeBILy-Gabon Trial. Int. J. Infect. Dis..

[25] Bustinduy A.L., Kolamunnage-Dona R., Mirochnick M.H., Capparelli E.V., Tallo V., Acosta L.P., Olveda R.M., Friedman J.F., Hope W.W. (2020). Population Pharmacokinetics of Praziquantel in Pregnant and Lactating Filipino Women Infected with *Schistosoma japonicum*. Antimicrob. Agents Chemother..

[26] Gray D.J., Ross A.G., Li Y.S., McManus D.P. (2011). Diagnosis and Management of Schistosomiasis. BMJ.

[27] McManus D.P., Gray D.J., Li Y., Feng Z., Williams G.M., Stewart D., Rey-Ladino J., Ross A.G. (2010). Schistosomiasis in the People’s Republic of China: The Era of the Three Gorges Dam. Clin. Microbiol. Rev..

[28] Park S.K., Gunaratne G.S., Chulkov E.G., Moehring F., McCusker P., Dosa P.I., Chan J.D., Stucky C.L., Marchant J.S. (2019). The Anthelmintic Drug Praziquantel Activates a Schistosome Transient Receptor Potential Channel. J. Biol. Chem..

[29] Adam I., Elwasila E., Homeida M. (2005). Praziquantel for the Treatment of *Schistosomiasis mansoni* during Pregnancy. Ann. Trop. Med. Parasitol..

[30] Adam I., Elwasila E.T., Homeida M. (2004). Is Praziquantel Therapy Safe during Pregnancy?. Trans. R. Soc. Trop. Med. Hyg..

[31] Salam R.A., Cousens S., Welch V., Gaffey M., Middleton P., Makrides M., Arora P., Bhutta Z.A. (2019). Mass Deworming for Soil-Transmitted Helminths and Schistosomiasis among Pregnant Women: A Systematic Review and Individual Participant Data Meta-Analysis. Campbell Syst. Rev..

[32] da Paz V.R.F., Sequeira D., Pyrrho A. (2017). Infection by *Schistosoma mansoni* during Pregnancy: Effects on Offspring Immunity. Life Sci..

[33] Gerstenberg J., Mishra S., Holtfreter M., Richter J., Davi S.D., Okwu D.G., Ramharter M., Mischlinger J., Schleenvoigt B.T. (2024). Human Placental Schistosomiasis—A Systematic Review of the Literature. Pathogens.

[34] Drugs.com Team Praziquantel Drug Interactions—Drugs.Com. https://www.drugs.com/drug-interactions/praziquantel-index.html.

[35] Zdesenko G., Mutapi F. (2020). Drug Metabolism and Pharmacokinetics of Praziquantel: A Review of Variable Drug Exposure during Schistosomiasis Treatment in Human Hosts and Experimental Models. PLoS Negl. Trop. Dis..

[36] Kiani J., Imam S.Z. (2007). Medicinal Importance of Grapefruit Juice and Its Interaction with Various Drugs. Nutr. J..

[37] Adam I., ALhabardi N.A., Al-Wutayd O., Khamis A.H. (2021). Prevalence of Schistosomiasis and Its Association with Anemia among Pregnant Women: A Systematic Review and Meta-Analysis. Parasites Vectors.

[38] Leenstra T., Acosta L.P., Langdon G.C., Manalo D.L., Su L., Olveda R.M., McGarvey S.T., Kurtis J.D., Friedman J.F. (2006). Schistosomiasis Japonica, Anemia, and Iron Status in Children, Adolescents, and Young Adults in Leyte, Philippines. Am. J. Clin. Nutr..

[39] Okoroafor C.F., Umeononihu O.S., Anikwe C.C., Adebayo A.J., Ezeigwe C.O., Olaleye A.A., Okorochukwu B.C., Ogelle O.M., Eleje G.U. (2024). Impact of Schistosomiasis Infection on Maternal and Neonatal Outcomes in Pregnancy: A Prospective Cohort Study in Nigeria. BMC Pregnancy Childbirth.

[40] WHO WHO Recommendations on Antenatal Care for a Positive Pregnancy Experience. https://www.who.int/publications/i/item/9789241549912.

[41] Owais A., Merritt C., Lee C., Bhutta Z.A. (2021). Anemia among Women of Reproductive Age: An Overview of Global Burden, Trends, Determinants, and Drivers of Progress in Low-and Middle-Income Countries. Nutrients.

[42] Faust C.L., Osakunor D.N.M., Downs J.A., Kayuni S., Stothard J.R., Lamberton P.H.L., Reinhard-Rupp J., Rollinson D. (2020). Schistosomiasis Control: Leave No Age Group Behind. Trends Parasitol..

[43] Hotez P.J., Harrison W., Fenwick A., Bustinduy A.L., Ducker C., Mbabazi P.S., Engels D., Kjetland E.F. (2019). Female Genital Schistosomiasis and HIV/AIDS: Reversing the Neglect of Girls and Women. PLoS Negl. Trop. Dis..

[44] King C.H., Dickman K., Tisch D.J. (2005). Reassessment of the Cost of Chronic Helmintic Infection: A Meta-Analysis of Disability-Related Outcomes in Endemic Schistosomiasis. Lancet.

[45] Colley D.G., Bustinduy A.L., Secor W.E., King C.H. (2014). Immunology of Human Schistosomiasis. Lancet.

[46] Pearce E.J., MacDonald A.S. (2002). The Immunobiology of Schistosomiasis. Nat. Rev. Immunol..

[47] Wilson S., Jones F.M., Kenty L.C., Mwatha J.K., Kimani G., Kariuki H.C., Dunne D.W. (2014). Posttreatment Changes in Cytokines Induced by *Schistosoma mansoni* Egg and Worm Antigens: Dissociation of Immunity- and Morbidity-Associated Type 2 Responses. J. Infect. Dis..

[48] Colt S., Jarilla B., Baltazar P., Tallo V., Acosta L.P., Wu H.W., Barry C.V., Kurtis J.D., Olveda R.M., Friedman J.F. (2021). Effect of Maternal Praziquantel Treatment for *Schistosoma japonicum* Infection on the Offspring Susceptibility and Immunologic Response to Infection at Age Six, a Cohort Study. PLoS Negl. Trop. Dis..

[49] Holtfreter M.C., Mischlinger J., Davi S.D., Schleenvoigt B.T. (2023). Investigation on Birth Weight Outcomes in Schistosomiasis and Praziquantel Research: A Correspondence. Eur. J. Med. Res..

[50] Tweyongyere R., Mawa P.A., Kihembo M., Jones F.M., Webb E.L., Cose S., Dunne D.W., Vennervald B.J., Elliott A.M. (2011). Effect of Praziquantel Treatment of *Schistosoma mansoni* during Pregnancy on Immune Responses to Schistosome Antigens among the Offspring: Results of a Randomised, Placebo-Controlled Trial. BMC Infect. Dis..

[51] Lo N.C., Bogoch I.I., Blackburn B.G., Raso G., N’Goran E.K., Coulibaly J.T., Becker S.L., Abrams H.B., Utzinger J., Andrews J.R. (2015). Comparison of Community-Wide, Integrated Mass Drug Administration Strategies for Schistosomiasis and Soil-Transmitted Helminthiasis: A Cost-Effectiveness Modelling Study. Lancet Glob. Health.

[52] Lo N.C., Lai Y.S., Karagiannis-Voules D.A., Bogoch I.I., Coulibaly J.T., Bendavid E., Utzinger J., Vounatsou P., Andrews J.R. (2016). Assessment of Global Guidelines for Preventive Chemotherapy against Schistosomiasis and Soil-Transmitted Helminthiasis: A Cost-Effectiveness Modelling Study. Lancet Infect. Dis..

[53] Wiegand R.E., Fleming F.M., de Vlas S.J., Odiere M.R., Kinung’hi S., King C.H., Evans D., French M.D., Montgomery S.P., Straily A. (2022). Defining Elimination as a Public Health Problem for Schistosomiasis Control Programmes: Beyond Prevalence of Heavy-Intensity Infections. Lancet Glob. Health.

[54] Rakotozandrindrainy R., Rakotoarivelo R.A., Kislaya I., Marchese V., Rasamoelina T., Solonirina J., Ratiaharison E.F., Razafindrakoto R., Razafindralava N.M., Rakotozandrindrainy N. (2024). Schistosome Infection among Pregnant Women in the Rural Highlands of Madagascar: A Cross-Sectional Study Calling for Public Health Interventions in Vulnerable Populations. PLoS Negl. Trop. Dis..

[55] Elliott A.M., Ndibazza J., Mpairwe H., Muhangi L., Webb E.L., Kizito D., Mawa P., Tweyongyere R., Muwanga M. (2011). Treatment with Anthelminthics during Pregnancy: What Gains and What Risks for the Mother and Child?. Parasitology.

[56] Bourke C.D., Nausch N., Rujeni N., Appleby L.J., Trottein F., Midzi N., Mduluza T., Mutapi F. (2014). Cytokine Responses to the Anti-Schistosome Vaccine Candidate Antigen Glutathione-S-Transferase Vary with Host Age and Are Boosted by Praziquantel Treatment. PLoS Negl. Trop. Dis..

[57] WHO Expert Committee (2002). WHO Technical Report Series 912 (2002)—Prevention and Control of Schistosomiasis and Soil-Transmitted Helminthiasis.

[58] Hu J.C. (1998). Praziquantel for treatment of acute and chronic schistosomiasis during pregnancy: A report of 4 cases. Parasit. Dis. Control Res..

[59] Yang H. (2002). Treatment and nursing for an acute schistosomiasis patient with pregnancy. Chin. J. Schisto Control.

[60] Yang L. (2007). Acute schistosomiasis combined with late gestation: One case report. Chin. J. Schisto Control.

[61] WHO (2024). WHO-PQ Recommended Summary of Product Characteristics.

[62] World Health Organization (2018). Assessing the Efficacy of Anthelminthic Drugs Against Schistosomiasis and Soil-Transmitted Helminthiases.

[63] Rollinson D., Knopp S., Levitz S., Stothard J.R., Tchuem Tchuenté L.A., Garba A., Mohammed K.A., Schur N., Person B., Colley D.G. (2013). Time to Set the Agenda for Schistosomiasis Elimination. Acta Trop..

[64] Bustinduy A.L., Friedman J.F., Kjetland E.F., Ezeamama A.E., Kabatereine N.B., Stothard J.R., King C.H. (2016). Expanding Praziquantel (PZQ) Access beyond Mass Drug Administration Programs: Paving a Way Forward for a Pediatric PZQ Formulation for Schistosomiasis. PLoS Negl. Trop. Dis..

[65] CDC Team Clinical Care of Schistosomiasis Schistosomiasi|CDC. https://www.cdc.gov/schistosomiasis/hcp/treatment/index.html.

